# Cancer-Associated Fibroblast Subgroups Showing Differential Promoting Effect on HNSCC Progression

**DOI:** 10.3390/cancers13040654

**Published:** 2021-02-06

**Authors:** Soo Hyun Kang, Su Young Oh, Heon-Jin Lee, Tae-Geon Kwon, Jin-Wook Kim, Sung-Tak Lee, So-Young Choi, Su-Hyung Hong

**Affiliations:** 1 Department of Microbiology and Immunology, School of Dentistry, Kyungpook National University, Daegu 700-412, Korea; black_bean@knu.ac.kr (S.H.K.); oohsuy@knu.ac.kr (S.Y.O.); heonlee@knu.ac.kr (H.-J.L.); 2 Department of Oral and Maxillofacial Surgery, School of Dentistry, Kyungpook National University, Daegu 700-412, Korea; kwondk@knu.ac.kr (T.-G.K.); vocaleo@knu.ac.kr (J.-W.K.); st0907@knu.ac.kr (S.-T.L.)

**Keywords:** head and neck squamous cell carcinoma, cancer-associated fibroblast, DNA microarray, collagen

## Abstract

**Simple Summary:**

It is generally accepted that fibroblasts represent a heterogeneous population of cells with different functions depending on the cell type. Although numerous reports have stated that cancer-associated fibroblast (CAF) promotes cancer progression, few studies have shown that they inhibit cancer progression. We propose that CAFs derived from some HNSCC patients is less effective in promoting cancer progression than CAFs from other patients and that specific collagen proteins may be involved in this process.

**Abstract:**

*Background*: The critical effect of the tumor microenvironment on cancer progression is well recognized. Recent research suggests that the cancer-promoting properties of the tumor stroma may be attributed to fibroblasts. However, the effect of cancer-associated fibroblast (CAF) on the progression of head and neck squamous cell carcinoma (HNSCC) is not well known. *Methods*: From the immunohistochemical analysis of head and neck squamous cell carcinoma (HNSCC) tissues, we divided CAF into two groups depending on the presence or absence of a well-demarcated boundary between epithelial cancer cells and the surrounding extracellular matrix (ECM). Primary culture of CAF was performed, followed by co-transplantation with HNSCC cells into mice oral mucosa, and the tumorigenesis was compared. The mRNA expression patterns between these two CAF groups were compared using DNA microarray analysis. *Results*: CAFs from cancer tissues that showed no demarcation between ECM and epithelial cancer cells (CAF-Promote) tended to stimulate Matrigel invasion of HNSCC cells. Conversely, CAFs from cancer tissues that showed a boundary with epithelial cancer cells (CAF-Delay) caused no remarkable increase in Matrigel invasion. Compared with CAF-P, CAF-D is less effective in promoting FaDu tumorigenicity in the mouse model. In DNA microarray analysis, *COL3A1* and *COL6A6* showed particularly high expression in the CAF-D group. *Conclusions*: These cancer stroma-derived collagen proteins might delay the HNSCC progression. These findings are expected to provide vital information for predicting HNSCC prognosis and developing drug targets in the future.

## 1. Introduction

Recent advancements in cancer biology have substantially changed the understanding of the functional significance of the tumor microenvironment. The tumor microenvironment plays a vital role in neoplastic cell tumorigenesis, progression, and metastasis [1,2]. Fibroblasts, one of the major cell components in the tumor microenvironment, are essential in tissue homeostasis and wound healing; further, it has been demonstrated that the fibroblasts in tumors (cancer-associated fibroblasts, CAFs) are key players in the process of tumorigenesis [3,4]. CAFs can be distinguished from neoplastic cells that have undergone epithelial–mesenchymal transition. However, the molecular definition of CAFs remains a debated topic. In case of tissue damage, fibroblasts proliferate and differentiate into myofibroblasts, a process characterized by the de novo expression of α-smooth muscle actin (α-SMA) and splice fibronectin variants [5,6]. Interestingly, myofibroblasts, the predominant CAFs, contribute to tumor progression [7]. CAFs secrete growth factors and chemokines that alter the extracellular matrix (ECM) and oncogenic signals, thereby increasing the proliferation and invasion of cancer cells [1,8]. Moreover, CAFs promote cancer progression in vivo when co-injected with tumor cells or are recruited to the tumor site [9,10]. Emerging data have demonstrated that fibroblasts represent a heterogeneous population of cells with different functions depending on the cell type [4]. Several studies have shown that normal tissue-derived fibroblasts inhibit the growth of tumor cells [11,12]. However, few studies have shown that fibroblasts adjacent to cancer tissues or CAFs inhibit cancer progression.

ECM is primarily a physical scaffold that binds cells and tissues together. It is typically accepted that cellular behavior and proper tissue function are mainly regulated by protein fibers, such as collagen, within ECM. ECM homeostasis, which is critical for organ function, is maintained via a delicate balance between ECM protein synthesis, protein fibril assembly, remodeling, and degradation [13]. Disrupting this balance results in various pathological conditions, thereby highlighting the correlation between altered ECM structure and disease grade [14]. Recent studies have shown that ECM affects cell fate by altering its degree of stiffness, organization, and molecular composition, thereby regulating cell migration and angiogenesis [15,16]. Furthermore, the ECM scaffold undergoes considerable structural changes during tumor progression, including increased collagen deposition and enhanced matrix cross-linking [17]. Considering this viewpoint, it is extremely interesting that breast cancer cell migration is dependent on collagen type I fibril alignment rather than stiffness [18] and that the collagen fibril diameter regulates cell morphology and invasiveness [19]. Interestingly, recent data have suggested that multiplex immunohistochemical (IHC) analyses of human pancreatic cancer stroma revealed a wide spectrum of phenotypical CAF variations in terms of protein expression levels of known fibroblastic markers such as α-SMA and fibroblast activation protein (FAP) [20]. In addition, they showed that well-differentiated tumor ducts were tightly surrounded by α-SMA-dominant CAFs with thick collagen bands. Furthermore, collagen-rich stroma was associated with prolonged cancer-specific survival compared with α-SMA- or FAP-rich stroma [20]. Brisson et al. showed that type III collagen (COL3) in the tumor microenvironment suppresses procarcinogenic collagen and myofibroblasts by regulating stromal organization [21]. Collagens, the most abundant protein in ECM, account for one-third of total proteins in humans; further, at least 28 different types of collagens have been identified in vertebrates [22]. According to the structural properties of ECM, collagens can be categorized into classical fibrillar and network-forming collagens, fibril-associated collagens with interrupted triple helices, membrane-associated collagens with interrupted triple helices, and multiple triple-helical domains and interruptions [22]. However, information about the effect of each collagen subtype on cancer progression is scarce.

It is increasingly evident that three-dimensional (3D) cell culture models are better than the traditional 2D monolayer culture due to improved cell–cell interactions, cell–ECM interactions, and cell populations and structures that resemble in vivo architecture [23]. Multicellular spheroids are one of the most commonly used models for 3D cell culture. In recent years, advances in 3D cell culture technology facilitated uniform culture sizes with high-throughput scale [24]. The use of 3D spheroids for constructing in vivo mouse tumor model reportedly enables more appropriate preclinical evaluation than an animal model constructed using dissociated two-dimensional (2D) cells [25,26]. In the present study, we compared the effect of primary CAFs on tumorigenesis in mouse formed by FaDu cells grown as 3D spheroids. We aimed to determine the differential role of CAFs derived from patients with HNSCC in vitro and in an in vivo model.

## 2. Results

### 2.1. Characteristics of HNSCC Tissues according to the Marginal Shape between CAF and Cancer Epithelial Cells

**Figure 1:**
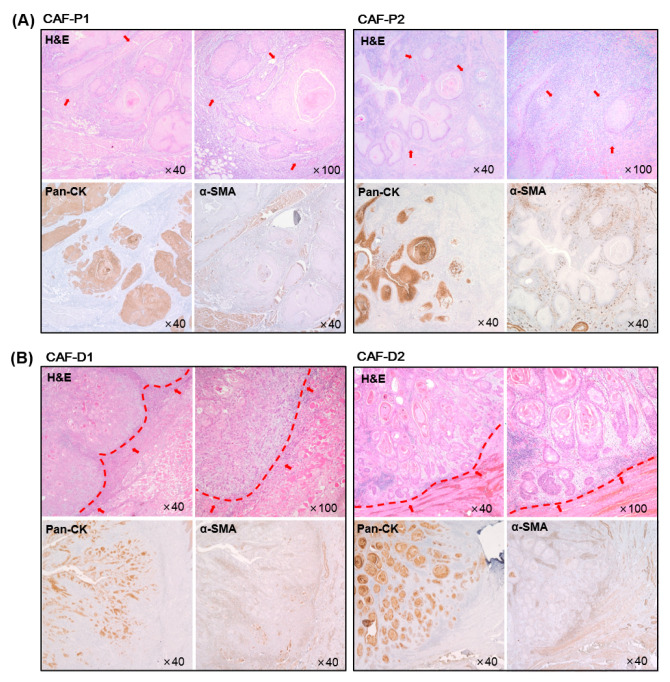
Immunohistochemical analysis of tumor tissues from HNSCC patients. Hematoxylin and eosin (H&E) staining was performed using HNSCC tumor tissues. Representative cancer stroma, including CAF, is shown with red arrows. Pan-cytokeratin (Pan-CK) and α-SMA staining was conducted at the same time to evaluate the epithelial cancer cells and CAFs, respectively. (**A**) Representative image for CAF-P. (**B**) Representative image for CAF-D. The boundary between epithelial cancer and adjacent stroma was marked with a red line.

On comparing the characteristics of the margin between ECM and cancer epithelial cells in HNSCC tissues, two characteristics were found. In one group (Figure 1A), the margin between cancer cells and CAF was unbounded. Conversely, the boundary between cancer cells and CAF was relatively demarcated in the other group (Figure 1B). In particular, the arrangement of CAF was such that it appeared to be surrounding cancer cells. Therefore, we named CAF of the former group as CAF-Promote or CAF-P and that of the latter group as CAF-Defense or CAF-D. CAF from each group was primary cultured to determine its effect on cancer progression.

### 2.2. Characterization of Fibroblast Cells Cultured from HNSCC Tissues

**Figure 2:**
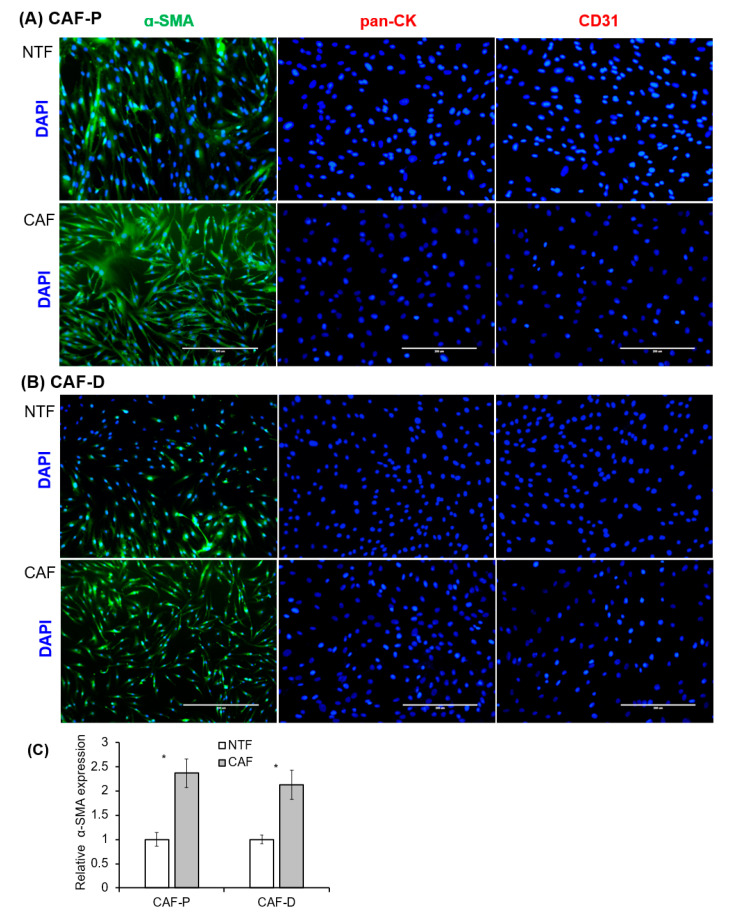
Immunocytochemical analysis of primary fibroblasts cultured from HNSCC patients’ tissues. (**A**,**B**) The same number of primary fibroblast cells after three passages were seeded in six-well plates containing cover slides and immunostained with antibodies specific for fibroblasts (α-SMA), epithelial cells (pan-CK), and endothelial cells (CD31). The white scale bar represents 200 μM. (**C**) α-SMA fluorescence was quantified by image analysis. Results represent the mean ± standard deviation of three experiments (* *p* < 0.05).

After 2–3 weeks of incubation, fibroblasts attached to the culture plate began to grow. All primary fibroblasts exhibited a homogeneous, spindle-shaped fibroblastic morphology. Fibroblasts that express α-SMA are considered the main constituents of the stroma in various cancers [27]. Immunocytochemical staining was performed with primary fibroblast cells cultured from four patients, and representative images from one patient are shown in Figure 2. To confirm epithelial and endothelial cell contamination within the isolated fibroblasts, we examined pan-cytokeratin (Pan-CK) and CD31 expression. The fibroblast cultures were negative for both these markers. Most of the CAF-P and CAF-D cells (>95%) exhibited positive staining for α-SMA. On the contrary, paired non-tumor fibroblast (NTF) cells showed weak-positive or negative signal by fluorescent α-SMA staining. α-SMA expression in primary fibroblasts was quantified by fluoresce image analysis. As shown in Figure 2C, there was no difference between CAF-P and CAF-D groups. However, α-SMA lever is significant higher in CAFs compared to paired NTFs. The expression patterns of these markers were maintained after several passages.

### 2.3. Effect of CAFs on Matrigel Invasion of HNSCC Cells

**Figure 3:**
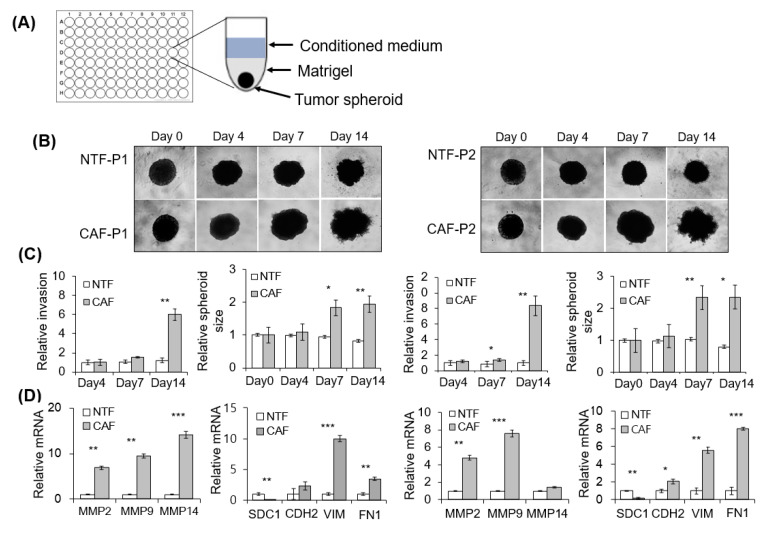

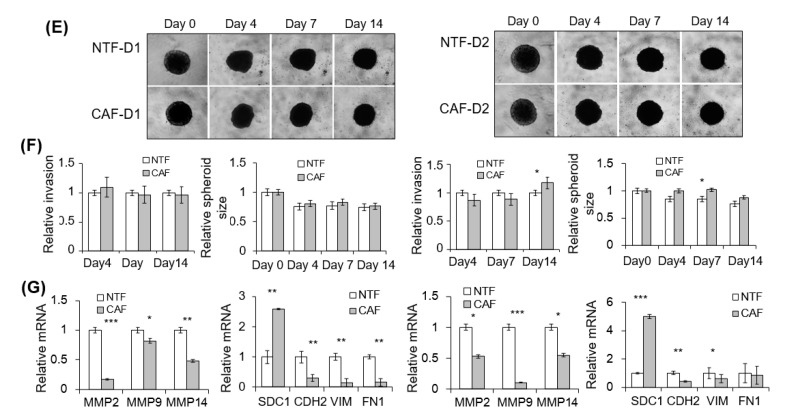
Effect of CAF-P and CAF-D on Matrigel invasion of FaDu spheroids. (**A**) To evaluate the effect of CAF-derived CM on 3D FaDu spheroid invasion, spheroid formation was performed in 96-well U-bottom Ultra-Low Attachment plate for two days (400 µm in diameter), followed by medium change with CM and Matrigel addition. (**B**) The spheroid invasion under CM from CAF-P was monitored for 14 days using phase-contrast microscopy (5× magnification). (**C**) Cell invasion was quantified by measuring the mean number of tube-like structures extending from the surface of each spheroid. The spheroid size was quantified using Cell^3^iMager. (**D**) MMPs mRNA expression in FaDu spheroids was analyzed using qPCR on day 14. mRNA expression of representative EMT markers was also compared at the same condition. (**E**–**G**) Same experiments were performed with CM derived from CAF-D. Results represent the mean ± standard deviation of 2–3 experiments (* *p* < 0.05, ** *p* < 0.01, *** *p* < 0.005).

We evaluated the effect of CAFs on 2D Matrigel invasion of HNSCC cells using a Transwell co-culture system (Figure S1A). Compared with CAF-D, the invasion of FaDu cells was significantly increased under co-culture with CAF-P (Figure S1B). The YD-10B cells showed similar pattern with the FaDu cells (Figure S1C). Furthermore, we investigated the effect of CAF-P- or CAF-D-derived condition medium (CM) on the invasive potential of 3D FaDu spheroids (Figure 3A). Considering the difference in CAF characteristics between patients, CAF-paired NTF was also compared. Corresponding differences were noticed in relative invasion (number of sprouts extending from spheroids) and spheroid size. No change was observed in spheroid invasion for four days; however, on day 14, Matrigel invasion by CAF-P-derived CM was enhanced compared with that by paired NTF-derived CM (Figure 3B). Corresponding differences were observed in the relative invasion (number of sprouts extending from spheroids) and FaDu spheroid size (Figure 3C). However, no significant change in Matrigel invasion or FaDu spheroid size was noted under the influence of CAF-D-derived CM for 14 days (Figure 3E,F). NTF-derived CM did not significantly affect Matrigel invasion and spheroid size after 14 days in both groups. Interestingly, MMPs mRNA expression was upregulated in FaDu spheroids co-cultured with CAF-P-derived CM compared with those co-cultured with paired NTF-derived CM (Figure 3D). However, MMP mRNA level remarkably decreased in FaDu spheroids co-cultured with CAF-D-derived CM compared with those co-cultured with paired NTF-derived CM (Figure 3G). Interestingly, mesenchymal markers such as N-cadherin (CDH2), vimentin (VIM), and fibronectin (FN1) showed similar mRNA expression pattern with MMPs at the same condition. Syndecan-1 (SDC1), one of the epithelial markers, showed opposite pattern with that of mesenchymal markers both in CAF-P- and CAF-D-derived CMs. (Figure 3D,G).

### 2.4. DNA Microarray Analysis of Primary CAF-P and CAF-D

**Figure 4:**
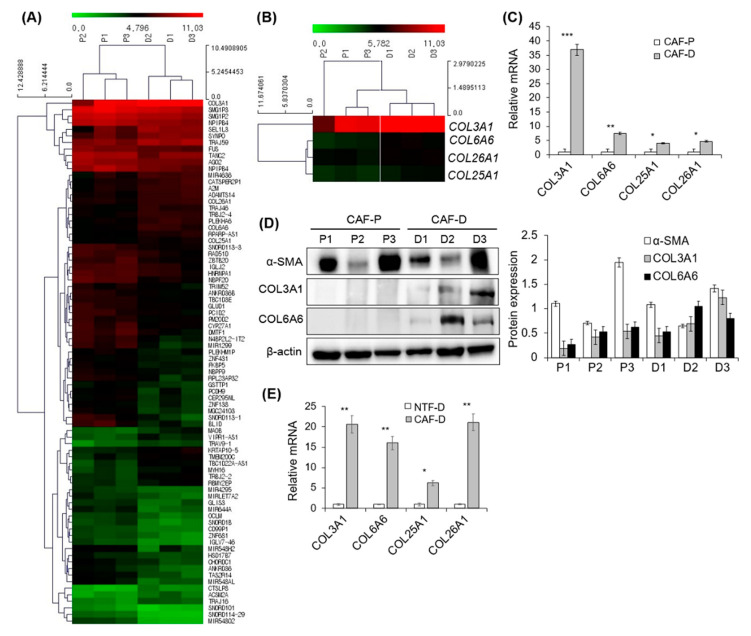
DNA microarray analysis of CF-P and CAF-D group. To evaluate the gene expression difference between CAF-P and CAF-D groups, microarray analysis with six samples of three primary fibroblasts in each group was performed with Affymetrix GeneChip^®^ Human Gene 2.0 ST array. (**A**) Heatmap of differentially expressed genes among the matched groups with a fold change of >1.75 and *p*-value of <0.05. (**B**) Heatmap of *COL3A1*, *COL6A6*, *COL25A1*, and *COL26A1* in two groups. (**C**) The mRNA expression levels of four collagen genes were compared using qPCR analysis in the CAF-P and CAF-D group fibroblasts. (**D**) Collagen protein expression was compared in the same sample groups. (**E**) mRNA expression of four COL genes was compared in four sets of CAF-D and paired NTF-D. Results represent the mean ± standard deviation of three experiments (* *p* < 0.01, ** *p* < 0.005, *** *p* < 0.0001).

Following quantile normalization of the raw data of DNA microarray, the expression profile was obtained from primary CAF cells in the two groups and submitted to the GEO repository (GSE160919). We identified differentially expressed genes among the matched groups with a fold change of >1.75 and *p*-value of <0.05. The heatmap of these genes is shown in Figure 4A. They consist of 27 upregulated genes and 53 downregulated genes. The identified gene list was uploaded to the online software DAVID for molecular function and biological process analyses. The results are shown in supplementary Table S2. Enrichment analysis related to the biological process and cellular compartment showed that collagen catabolic process and collagen trimer were the significant functional annotation terms (*p* < 0.05). The search for different collagen mRNAs in our microarray data revealed that COL25A1 and COL26A1 showed 1.43–1.49 times higher expression in the CAF-D group than in the CAF-P group (*p* < 0.05). The fold ratio of each collagen protein in CAF-D versus CAF-P is shown in Table S3. David functional annotation of collagen proteins is shown in Table S4. 

Figure 4B shows a heatmap of COL3A1, COL6A6, COL25A1, and COL26A1. Collagen mRNA expression level was confirmed in each of the four primary CAF-P and CAF-D samples by qPCR. As shown in Figure 4C, COL3A1, COL6A6, COL25A1, and COL26A1 mRNA expression levels were significantly higher in the CAF-D group than in the CAF-P group. In addition, COL3A1 and COL6A6 protein expression levels were increased in the CAF-D group under the same condition (Figure 4D). There was no significant difference of α-SMA expression in DNA microarray or western blot analysis. Because the characteristics of CAF-D resemble NTF in that it can delay or inhibit cancer progression, we compared the expression of four COL genes in four sets of CAF-D and NTF-D. As shown in Figure 4E, mRNA expression of COL genes in CAF-D group was significantly higher than in NTF-D group, suggesting that CAF-D is differentiated from NTF. 

### 2.5. IHC Analysis of Collagen Proteins in Tissues Obtained from Patients with HNSCC

**Figure 5:**
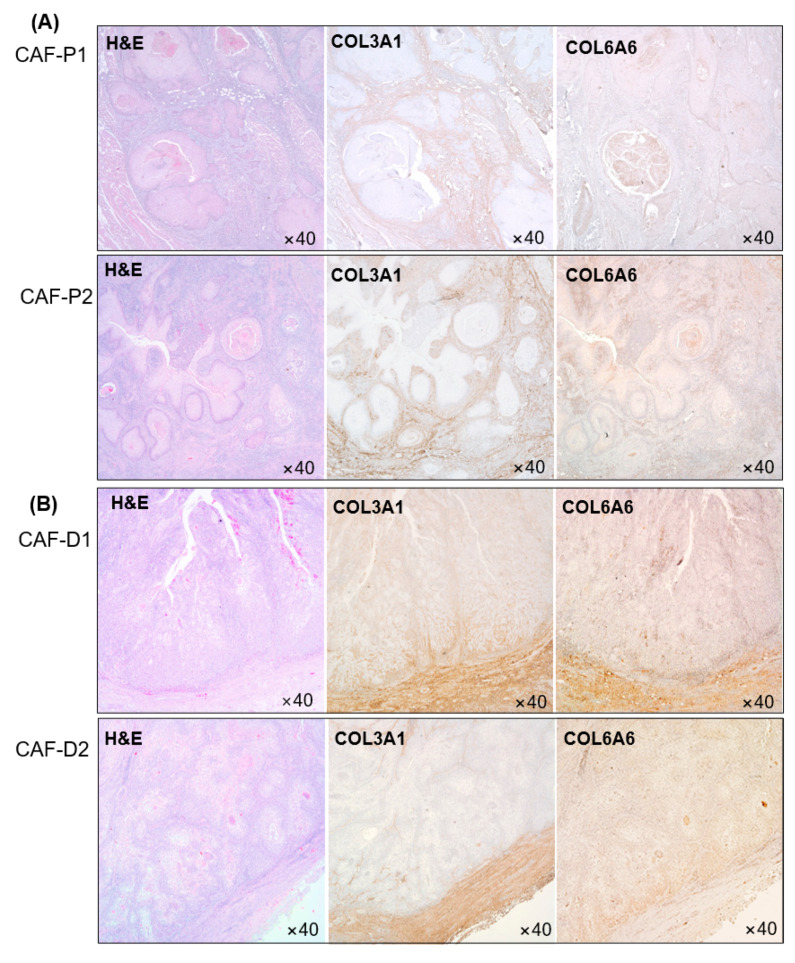
Collagen protein expression in HNSCC patients’ tissues. COL3A1 and COL6A6 protein expression was evaluated in CAF-P (**A**) and CAF-D (**B**) tissues from patients with HNSCC.

This experiment was performed with tissues collected from four patients, and two sets of representative images are shown in Figure 5. COL3A1 and COL6A6 protein expression levels were higher in the CAF-D group than in the CAF-P group. CAF-P showed a weak positive staining pattern (Figure 5A). Conversely, as shown in Figure 5B, COL3Al and COL6A6 in CAF-D showed more amount of staining along the border of cancer epithelial cells. 

### 2.6. Mouse Tumorigenesis from FaDu and CAF-P/CAF-D

**Figure 6:**
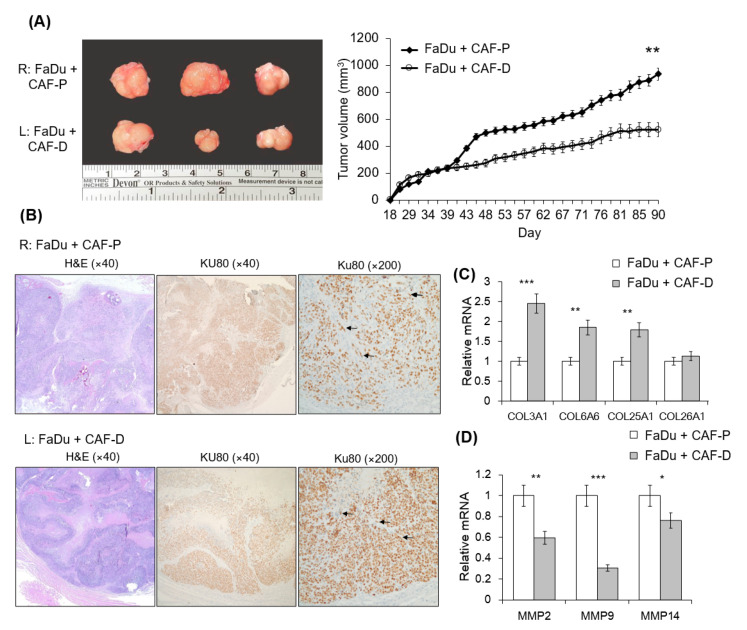
Mouse xenograft tumor formation from FaDu spheroids with CAF-P or CAF-D. FaDu spheroids (<400 μm in diameter) were prepared in 96-well U-bottom Ultra-Low Attachment plates. Overall, 50 FaDu spheroids (approximately 5 × 10^5^ cells) were co-injected with the same number of CAF-P and CAF-D cells into the right and left cheeks of the mouse oral mucosa, respectively. (**A**) Representative images show the appearance of tumors after sacrifice. At 90 days after injection, tumor size was measured using a caliper. (**B**) Immunohistochemical analysis of mice tumor tissues was performed with the Ku80 antibody to evaluate whether they are derived from FaDu and human fibroblasts. Representative Ku80-positive cells are indicated with black arrows. (**C**,**D**) Collagen and MMP mRNA expression levels in mice tumor tissues were compared. (* *p* < 0.05, ** *p* < 0.01, *** *p* < 0.005).

We compared tumorigenesis in the CAF-P and CAF-D groups upon injection of FaDu spheroids in the oral mucosa of the right (CAF-P) or left (CAF-D) cheek of mice. Interestingly, FaDu-derived tumors co-injected with CAF-P were significantly larger than those co-injected with CAF-D (Figure 6A). Figure 6B shows that human-specific Ku80 biomarkers were widely stained in tumor tissues, including epithelial cells and fibroblasts, suggesting that FaDu and CAF cells injected into the mice cheeks were well involved in xenograft tumor formation. FaDu-derived mice tumor tissues co-injected with CAF-D showed higher *COL3A1*, *COL6A6*, *COL25A1*, and *COL26A1* expression levels than those co-injected with CAF-P (Figure 6C). Interestingly, *MMP2*, *MMP9*, and *MMP14* mRNA expression was opposite to that of the collagen mRNA level (Figure 6D).

### 2.7. Effect of CAF-D with Collagen Knockdown on FaDu Matrigel Invasion

**Figure 7:**
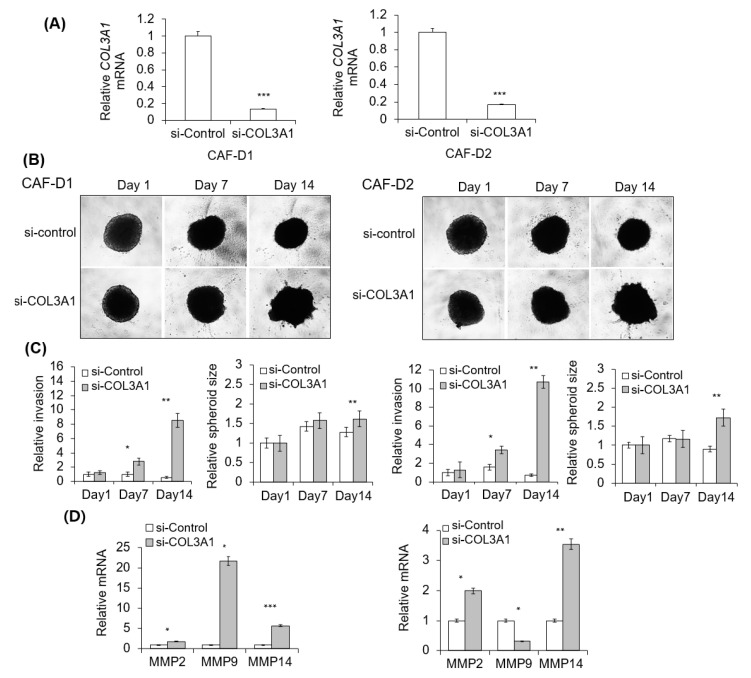
Effect of collagen knockdown-CAF-D on FaDu spheroid invasion. (**A**) CAF-D cells were transfected with siCOL3A1 for two days, and CM was collected. siRNA efficiency was evaluated using qPCR analysis. (**B**) FaDu spheroid (400 μm in diameter) was formed by culturing a 96-well U-bottom Ultra-Low Attachment plate for two days. CM and Matrigel were added to each well, and the spheroid invasion was monitored for 14 days using phase-contrast microscopy (5× magnification). (**C**) Cell invasion was quantified by measuring the mean number of tube-like structures extending from the surface of each spheroid. The spheroid size was quantified using Cell^3^iMager. (**D**) MMPs mRNA expression in FaDu spheroids were analyzed using qPCR on day 14. Results represent the mean ± standard deviation of three experiments (* *p* < 0.05, ** *p* < 0.01, *** *p* < 0.005).

We investigated the effect of CAF-derived CM transfected with siCOL3A1 or siCOL6A6 on the invasive potential of FaDu spheroids. Figure 7A shows the efficiency of gene knockdown with siCOL3A1 mixture treatment for two days. As shown in Figure 7B, no change was observed in spheroid invasion for 14 days in FaDu spheroids under CAF-D-derived CM transfected with the control siRNA. However, the invasion was enhanced by COL3A1-knockdown-CAF-D-derived CM after 14 days. Corresponding differences were noticed in relative invasion and spheroid size (Figure 7C). As shown in Figure 7D, MMPs mRNA expression was upregulated in FaDu spheroids by CM derived from collagen-knockdown-CAF-D. Unfortunately, CM from siCOL6A6-transfected CAF-D induced nonspecific cell death of FaDu spheroid. Therefore, we investigated the effect of siCOL6A6 on 2D HNSCC cells invasion with Transwell co-culture system. The results for FaDu and YD-10B cell lines were presented in supplementary Figure S2. Specific gene knockdown with siCOL3A1 or siCOL6A6 in CAF-D cell accelerated Transwell Matrigel invasion of 2D HNSCC cells as compared with siControl-transfected CAF-D cells (Figure S2).

### 2.8. Survival Analysis of COL Genes in HNSCC Patients

**Figure 8:**
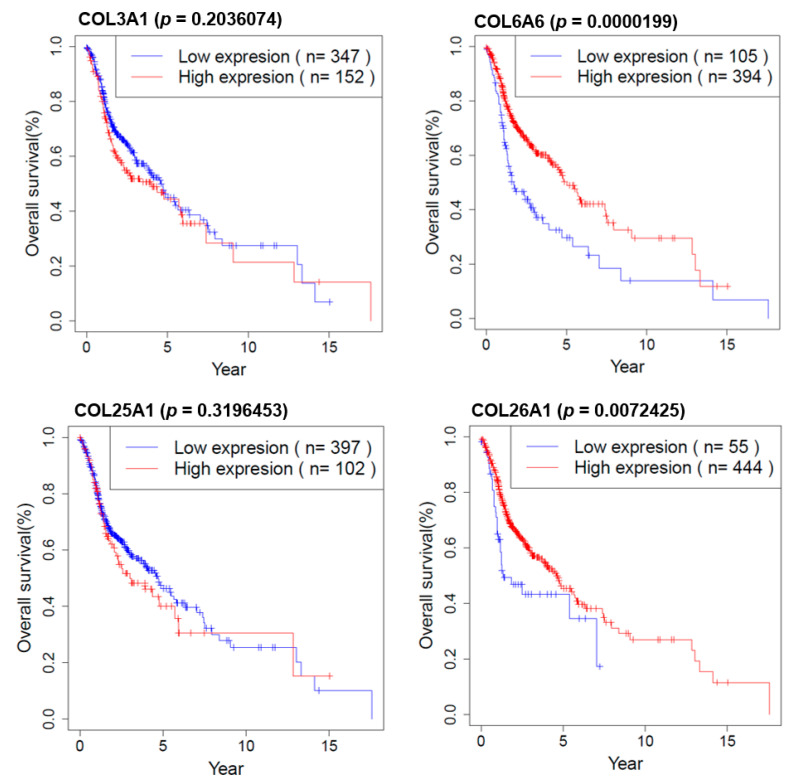
Kaplan–Meier survival plots for HNSCC patients according to COL genes.

We investigated the effect of COL genes on the survival of HNSCC patients in R (version 3.3.1). Overall survival information is downloaded from TCGA (The Cancer Genome Atlas). As shown in Figure 8, *COL6A6* and *COL26A1* are strongly associated with longer survival according to the Kaplan–Meier survival analysis.

## 3. Discussion

Although most of the fibroblasts derived from normal tissues are known to inhibit tumor cell proliferation, some have shown a stimulatory effect on tumor cell growth. The proportion of inhibitory and stimulatory fibroblasts differed depending on the target tumor cells [12]. Therefore, it is believed that CAFs and CAF-derived factors differentially act in a cellular context-dependent fashion and that the intrinsic properties of CAFs determine their tumor-promoting and/or tumor-inhibiting activities. Augsten suggested that fibroblast activities are regulated by their intrinsic expression program related to the fibroblast site of origin and modulated by external signals [28].

CAFs express various factors that contribute to shaping the environment, including pro-tumorigenic factors or factors that suppress the action of tumor-resident cells [28]. In the present study, cases with different arrangement patterns between tumor cells and surrounding fibroblast cells were frequently found on observing the tumor tissue slides of patients with HNSCC. The first is the case where there has been no obvious boundary between fibroblast cells and tumor tissue. In the remaining groups, the fibroblast cells appeared to surround the tumor cells, wherein the boundaries of the tumor cells were well demarcated. In the present study, we have revised whether there is a conflicting effect of CAFs on the progress of human HNSCC between these two CAF groups.

Augsten suggested that CAF can be polarized toward cells that display tumor inhibitory activity or different types of cells with tumor-promoting activity [28]. However, no previous data describe the molecular mechanisms underlying the differential effect of CAF on cancer progression in the tumor microenvironment. Therefore, a better phenotypic and functional characterization of CAFs is required to elucidate their biological role. Chang et al. showed that CAFs expressing the ligand Slit2 inhibited the tumorigenicity of breast cancer cells expressing the corresponding Robo1-receptor on their surface, thereby suggesting Slit2 as a potential effector of this tumor-inhibitory CAF-subtype [29]. Furthermore, Slit expression was reported to have prognostic significance predicting overall survival and metastasis occurrence. Green et al. observed that fibroblast-derived Wnt3a could promote or inhibit the growth of different orthotopically growing patient-derived breast xenograft tumors [30]. However, the molecular basis for the opposing behavior of Wnt3a-expressing fibroblasts remained unelucidated. However, these recent studies have provided initial experimental evidence that the same CAF type can exert a broader spectrum of activities ranging from tumor stimulation to tumor inhibition. Furthermore, because the action of CAFs in the tumor microenvironment is dependent on the nature of the interacting cell type, identification of the distinct CAF activation states would be of importance in future studies.

The progressive ECM remodeling produces typical morphological changes with significant impact on tumor cell biology, including gene expression, cell differentiation, proliferation, migration, and treatment responses [31]. Collagen affects the tumor microenvironment by regulating ECM remodeling and promotes tumor infiltration, angiogenesis, invasion, and migration [32]. Although collagen was conventionally considered as a passive barrier to resist tumor cells [33], it is now evident that collagen is also actively involved in promoting tumor progression [32]. However, according to the recent results of Ogawa et al. regarding the distinct stroma types based on stromal heterogeneity, patients with collagen-rich cancer stroma showed decreased MMP gene expression levels with longer survival compared with other patients with cancer stroma highly expressing CAF markers such as α-SMA or FAP [20]. We observed that the fibroblast CAF-D group appeared to act as a physical barrier that tightly surrounds the cancer cells and protects from them expanding to the surrounding. Owing to this effect of these primary cultured CAF cells on the growth and Matrigel invasion of co-cultured FaDu spheroid, the spheroid size and sprout formation of FaDu with CAF-D were significantly decreased compared with those with CAF-P. Furthermore, similar results were confirmed by in vivo tumorigenesis following the implantation of FaDu spheroids and CAF-P and CAF-D cells into the cheeks of nude mice. Moreover, CM cultured for two days after *COL3A1* knockdown with specific siRNA in CAF-D cells significantly increased Matrigel invasion of FaDu spheroids.

Our DNA microarray analysis conducted to compare mRNA expression patterns of the CAF-D and CAF-P groups showed that the expression levels of several collagen proteins in the CAF-D group were significantly increased. In particular, *COL3A1*, *COL6A6*, *COL25A1*, and *COL26A1* expression levels were remarkably increased in the CAF-D group than those in the CAF-P group. Table S1 shows the information of patients participated in this study. There seems to be a correlation of CAF type with tumor stage. The tumor stage of the CAF-D group patients so far might be low. Unfortunately, the number of CAF-D samples is not enough to discuss this point, which is why retrospective study is needed. Interestingly, increased expression of *COL6A6* and *COL26A1* in HNSCC tissues is significantly associated with patients’ survival. A broader correlation of the gene signature of CAF-D and CAF-P with survival, as well as tumor stage including each individual collagen gene would be important to evaluate the significance of the findings. In addition, the phenotype of CAFs could be the result of the tumor stage, meaning that CAFs have co-evolved with cancer to have a more aggressive phenotype. 

Interestingly, Ohlund D. et al. showed that inflammatory CAFs (iCAF) form a pro-tumoral population and facilitate invasion and metastasis, as well as immune suppression [34]. On the contrary, myofibroblasts (myCAF) are probably involved in stromal and endothelial growth factors secretion [35]. When comparing genes typically expressed in iCAF and myCAF with our microarray data, CAF-P had commonalities with iCAF and CAF-D with myCAF. Many of the genes upregulated in iCAF showed lower expression in CAF-D (e.g., *PRDM1*, *LIF*, *CXCL1*, *CXCL2*, *CXCL12*, *KCNJ15*, *PRDM1*). In addition, many genes upregulated in myCAF showed increased expression in CAF-D (e.g., *CDH10*, *COL2A1*, *HCK*, *TAGLN*, *POSTN*, *HOPX*, *TPM2*, *CDH3*, *SHANK2*). In this study, we could not comment on other features related with iCAF or myCAF, seeing only the effect of CAF on the migration and invasion of cancer cells. Further study for a subsequent comparison on the characteristics of iCAF and myCAF with our CAF-P or CAF-D needs to be performed. 

Friedi et al. suggested that the reciprocal reprogramming of the tumor cells and the surrounding tissue structures in the cancer microenvironment contribute to the generation of diverse cancer invasion routes, enhanced tumor heterogeneity, and ultimately, sustained metastatic dissemination [36]. Previous data have shown that tumor cells migrate along bundled collagen tracks [37]. Collagen surrounding normal epithelial structures in breast tissue is typically curly and smooth. However, parallel with tumor development, collagen progressively thickens and linearizes, promoting metastasis by fostering cell migration into ECM. Indeed, a previous study showed that breast cancer cells and leukocytes rapidly migrate along collagen fibers [38]. In addition, Wang et al. showed that upregulated *COL3A1* in epithelial—but not stromal—cells predicted poor survival [39]. *COL3A1* is a member of the collagen family and is mainly expressed in extensible connective tissues, including skin and vessels. Reportedly, *COL3A1* was substantially overexpressed in the liver invasion front of the colorectal liver metastases compared with the tumor center and the normal tissues, suggesting a potential role of this gene in metastasis [40]. Conversely, Beck et al. showed that robust stromal expression of COL3 correlates with improved survival of patients with breast cancer [41]. Theocharidis showed that normal fibroblasts cultured on cell-derived matrices lacking COL6 displayed increased cell spreading, migration, and persistence [42]. Furthermore, in pituitary adenoma tissues, *COL6A6* reportedly inhibited cancer progression by inhibiting the PI3K-AKT pathway [43]. Despite these findings, the functional roles and mechanism of CAF-derived *COL3A1* and *COL6A6* in tumorigenesis are yet to be elucidated. However, our results support previous data that collagen would be a passive barrier to resist tumor cells [33].

CAFs are found in almost all solid tumors; however, there is no consensus on their molecular definition [44,45]. Although several markers have been suggested to define CAFs, these markers do not mark all CAFs and most markers are not even unique to CAFs or the fibroblasts lineage. The α-SMA is a robust CAF marker, which typically identifies CAFs with myofibroblast morphology [46]. However, α-SMA is also expressed by normal fibroblasts [47], and in some cases, normal fibroblasts show similar or higher α-SMA expression levels than CAFs [48]. Further, α-SMA expression is detected in other cell types, such as pericytes and smooth muscle cells surrounding vasculature, visceral smooth muscle cells, and cardiomyocytes [49]. The fibroblast-specific protein-1 (FSP-1) is another common marker for CAFs in several carcinomas. However, FSP-1 is not restricted to CAFs alone and is also expressed by epithelial cells undergoing epithelial–mesenchymal transition [50]. Therefore, currently, there are few markers that completely and exclusively define CAFs, which should be considered when interpreting results from different models. In the present study, IHC results in cancer tissues from patients with HNSCC showed that collagen protein stained only fibroblast cells differently from epithelial cancer cells. In the future, it is important to consider collagen as a CAF marker that improves accuracy through wider trials. 

## 4. Materials and Methods

### 4.1. Ethical Statement

Human tissue specimens were used after receiving written, informed consent from patients and approval from the Institutional Research Ethics Committee of Kyungpook National University Hospital (KNUH201704011, dated 11 May 2017) in accordance with the basic principles of the Declaration of Helsinki. All experimental protocols using mice followed the ARRIVE guidelines (Animal Research: Reporting of In Vivo Experiments) and were approved by the Animal Ethics Committee of Kyungpook National University (KNU 2017-51-2, dated 11 August 2017).

### 4.2. Chemicals and Reagents

Dulbecco’s Modified Eagle’s Medium (DMEM), RPMI-1640 medium, fetal bovine serum (FBS), and penicillin–streptomycin solution were obtained from Invitrogen (Carlsbad, CA, USA). Qiazol was obtained from Qiagen (Valencia, CA, USA), and 2× SYBR Green PCR Master Mix was obtained from Takara Bio (Otsu, Japan). HRP-conjugated anti-*β*-actin (Santa Cruz Biotechnology Cat# sc-47778HRP, RRID: AB_2714189) antibody was obtained from Santa Cruz Biotechnology (Santa Cruz, CA, USA). Rabbit anti-α-SMA (Abcam Cat# ab5694, RRID: AB_2223021), rabbit anti-CD31 (Abcam Cat# ab28364, RRID: AB_726362), and mouse anti-pan-cytokeratin (Abcam Cat# ab7753, RRID: AB_306047) antibodies were from Abcam (Cambridge, MA, USA). Rabbit anti-COL3A1 antibody (Proteintech Group Cat# 22734-AP-1) was obtained from Proteintech (Rosemont, IL, USA). Rabbit anti-COL6A6 (Thermo Fisher Scientific Cat# PA5-60958, RRID: AB_2640027) antibodies were acquired from Thermo Fisher Scientific, Inc. (Irvine, CA, USA). Rabbit anti-Ku80 (Cell Signaling Technology Cat#2753) antibody was obtained from Cell Signaling Technology (Danvers, MA, USA).

### 4.3. Immunohistochemical Analysis of Clinical Specimens

Paraffin-embedded tissue blocks were obtained from patients with HNSCC who underwent tumor resection for oral cancer treatment from 2017 to 2018 at the Kyungpook National University Dental Hospital. Patient information is presented in supplementary Table S1. After dewaxing, sections were blocked for 5 min, followed by incubation for 2 h at room temperature with pan-cytokeratin (1:500), COL3A1 antibody (1:500), or COL6A6 antibody (1:500). IHC staining was performed using the UltraTek Horseradish Peroxidase (HRP) Anti-olyvalent Kit (ScyTek Laboratories, Logan, UT, USA); the chromogen used was 3,3-diaminobenzidine (Dako, Carpinteria, CA, USA). Nuclei were counterstained with hematoxylin.

Tumor tissues were stained with Ku80 to evaluate whether mice xenograft tumor tissues were derived from injected human cells. Ku80 is human-specific and broadly expressed throughout the human body with no or low crossreactivity toward rat or mouse tissues [51].

### 4.4. Fibroblast Primary Culture from Fresh HNSCC Tissues

Tumor tissues for fibroblast culture were obtained by surgical resection from patients with HNSCC at Kyungpook National University Dental Hospital. None of the patients had undergone chemoradiotherapy before surgery. The stroma adjacent to the tumor mass was carefully separated by a pathologist, cut into the smallest possible sections in sterile DMEM, and then seeded into 10-cm culture dishes supplemented with 10% FBS. Tissue samples for NTF culture was collected from an area at least 1 cm away from the tumor. After 2–3 weeks, fibroblast cells were cultured in a six-well plate with a cover slide for immunocytochemical analysis. On the following day, the cells were washed and immediately fixed in 4% paraformaldehyde for 1 h. After washing, cells were blocked using albumin serum for 1 h at room temperature. Cells were immunostained with primary antibody for 16 h at 4 °C and then incubated with fluorescent secondary antibody at room temperature. Fluorescence images were observed under a fluorescence microscope (Carl Zeiss, Thornwood, NY, USA).

To obtain CM from CAFs, primary fibroblasts that have undergone three passages were grown for 48 h until they attained 80% confluence. The culture medium was collected and passed via a 0.45-μM pore filter; the filtrate was used as the CM. Cells from passages 3–6 were used for all experiments.

### 4.5. HNSCC Cell Culture and FaDu Spheroid Formation

The FaDu human HNSCC cell line (ATCC Cat# HTB-43, RRID: CVCL_1218) was obtained from American Type Culture Collection (Manassas, VA, USA) and cultured in DMEM containing 10% FBS and 1% penicillin–streptomycin solution at 37 °C in a 5% CO_2_ humidified atmosphere. The YD-10B cell line was cultured in the RPMI medium under the same conditions. Every two months, the cell lines were tested for contamination using the CellSafe Mycoplasma PCR Detection Kit (Cat# CS-D, CellSafe Co., Yongin, South Korea). To obtain FaDu spheroid, cells were seeded into a 96-well U-bottom Ultra-Low Attachment plate (4000 cells/well) (Corning Inc., Corning, NY, USA) and cultured for 2–3 days until spheroid formation (approximately 400 μM in diameter). YD-10B cells did not form spheroids.

### 4.6. Matrigel Invasion Assay

The 2D HNSCC cell invasion was evaluated in a co-culture system using Matrigel-coated 8.0-μm filter chambers (BD Biosciences, San Jose, CA, USA). CAF-P or CAF-D cells were seeded in a 24-well plate. HNSCC cells were resuspended in the medium, and 300-µL aliquots were added to Matrigel-coated transwell chambers. After culturing for 48–72 h, the cells were stained with 0.2% crystal violet in 10% ethanol; cells on the upper side of the transwell chamber were removed using a cotton swab, whereas those that migrated to the lower surface of the chamber were counted. The invasion index was calculated as the fold change in the number of invaded cells in the experimental group compared with that in the control group. Furthermore, we evaluated the effect of CAF-P- or CAF-D-derived CM on the Matrigel invasion of FaDu spheroids. Following the formation of FaDu spheroids on a 96-well U-bottom ultra-low attachment plate for two days, the medium was replaced with 100 μL CM, and 50 μL Matrigel was added to each well to provide a semi-solid matrix. When the Matrigel solution solidified, an additional 100 μL CM was added to prevent drying. Matrigel invasion was monitored for 14 days using phase-contrast microscopy (5× magnification) and quantified by measuring the mean number of tube-like structures extending from the surface of each spheroid. Furthermore, the spheroid diameter was measured to evaluate spheroid size using Cell3iMager (CC-5000; Screen Holdings Co., Kyoto, Japan).

### 4.7. Real-Time Polymerase Chain Reaction

Total RNA extraction, cDNA synthesis, and gene expression normalization were performed following standard protocols. The forward and reverse primers used for real-time quantitative polymerase chain reaction (qPCR) were as follows: *MMP2*, 5′-AGCTGCAACCTGTTTGTGCTG-3′ and 5′-CGCATGGTCTCGATGGTATTCT-3′; *MMP9*, 5′-ACGACGTCTTCCAGTACCGAGA-3′ and 5′-TAGGTCACGTAGCCCACTTGGT-3′; *MMP14*, 5′-CGAGGTGCCCTATGCCTAC-3′ and 5′-CTCGGCAGAGTCAAAGTGG-3′; *COL3A1*, 5′-TTGGGATTGCTGGGATCACT-3′ and 5′-TGGTTTCCCACTTTCACCCT-3′; *COL6A6*, 5′-CCACCTCACAGCTTACTCCA-3′ and 5′-CTCAGGAGAAAGGGCAGAC-3′; COL25A1, 5′-CATGTGGCCACTTTGGTTGA-3′ and 5′-TTGGTCACATGAGAGGCAGT-3′; *COL26A1*, 5′-GACCCACCTACAGAGTGTCC-3′ and 5′-GGTGCAGTTCATGCATTCCT-3′; *SDC1*, 5′-CGAATCTCTGTGCCTTCGTC-3, and 5′-GGAGCTTGGCAACACAGAAA-3′; *CDH2*, 5′-GGTGGAGGAGAAGAAGACCAG-3′ and 5′-CGCATCAGGCTCCACAGT-3′; *VIM*, 5′-GCAAAGCAGGAGTCCACTGAGT-3′ and 5′-ATTTCACGCTCTGGCGTTC-3′; *FN1*, 5′-CAGGATCACTTACGGAGAAACAG-3′ and 5′-GCCAGTGACAGCATACACAGTG-3′; and glyceraldehyde 3-phosphate dehydrogenase (*GAPDH*), 5′-AGATCATCAGCAATGCCTCCTG-3′ and 5′-CTGGGCAGGGCTTATTCCTTTTCT-3′. Gene expression levels were normalized to that of the housekeeping gene GAPDH. qPCR was conducted using the ABI 7500 real-time PCR system (Applied Biosystems, Foster City, CA, USA) in triplicate. Calculations were performed based on Δcycle threshold (ΔCt) values, which were determined by normalizing the mean Ct value of each sample to that of the endogenous GAPDH control and then calculating the 2^−ΔΔCt^ value.

### 4.8. DNA Microarray Analysis

To analyze the difference in gene expression levels between CAF-P and CAF-D groups, we attempted microarray analysis using six samples of three primary fibroblasts in each group with the Affymetrix GeneChip^®^ Human Gene 2.0 ST array (Affymetrix, Santa Clara, CA, USA) containing 40,716 gene-level probe set. Total RNA was isolated using Trizol reagent (Invitrogen). RNA quality was assessed by Agilent 2100 bioanalyzer (Agilent Technologies, Santa Clara, CA, USA), and the quantity was determined using the ND-1000 spectrophotometer (NanoDrop Technologies, Wilmington, DE, USA). The RNA sample was used as input into the Affymetrix procedure, as per the recommended protocol. Briefly, total RNA from each sample was converted to double-stranded cDNA using a random hexamer incorporating a T7 promoter. Amplified RNA (cRNA) was generated from the double-stranded cDNA template via an in vitro transcription (IVT) reaction and purified using the Affymetrix sample cleanup module. Thereafter, the cDNA was fragmented by UDG and APE 1 restriction endonucleases and end-labeled via a terminal transferase reaction incorporating a biotinylated dideoxynucleotide. Fragmented end-labeled cDNA was hybridized to the Affymetrix arrays and stained using streptavidin–phycoerythrin conjugate. Affymetrix array was scanned using Affymetrix Model 3000 G7 scanner, (Affymetric Inc., Santa Clara, CA, USA) and the image data was extracted through Affymetrix Command Console Software 1.1 (Affymetric Inc., Santa Clara, CA, USA). Data mining and graphic visualization were performed using ExDEGA software (Ebiogen Inc., Seoul, Korea).

### 4.9. Western Blot Analysis

Total protein was extracted, and the concentration was assayed as described previously [52]. Equal amounts of protein (30–40 µg) were separated using 8–10% sodium dodecyl sulfate-polyacrylamide gel electrophoresis (SDS-PAGE) and then transferred to a nitrocellulose membrane. After blocking with 5% skim milk for 30 min, the membrane was incubated overnight at 4 °C with primary antibodies; β-actin served as the loading control. HRP-conjugated secondary antibodies at 1:5000 dilutions were applied for 1 h at room temperature, and the blot was washed thrice in Tris-buffered saline containing 0.1% Tween 20. Protein bands were detected by enhanced chemiluminescence.

### 4.10. Mouse Xenograft Model

We evaluated the effect of CAF-P and CAF-D on FaDu spheroid-derived tumor growth in xenograft mice (six-week-old female BALB/c; Hyochang Science, Daegu, Korea). FaDu spheroids were prepared in 96-well U-bottom ultra-low attachment plates (<400 µm in diameter). Overall, 50 FaDu spheroids and 5 × 10^5^ CAFs were co-injected into five mice using a 22-gauge needle. To reduce the error caused by variability among mice, CAF-P and CAF-D were co-injected with FaDu spheroids into the oral mucosa of the right and left cheeks, respectively. After 90 days, mice were sacrificed, and tumor volume was measured using a clipper. Tumor histology was examined by IHC analysis.

### 4.11. Transfection of Small Interfering RNA

Primary CAF-D cells were transiently transfected with a small interfering RNA (siRNA) mixture comprising 2–3 specific oligonucleotides targeting the COL3A1 or COL6A6 transcript (siCOL3A1; Santa Cruz Biotechnology, siCOL6A6; ORIGENE). The cells (1 × 10^5^) were seeded on a 60-mm plate; on the following day, the medium was replaced with a serum-free medium immediately before transfection with siRNA at a final concentration of 10 nM using Lipofectamine 3000 (Thermo Fisher Scientific, Waltham, MA, USA). After 6 h, the medium was replaced with fresh serum-containing medium, followed by Matrigel invasion of HNSCC cells under the Transwell co-culture system. CM was collected from siRNA-transfected CAFs after two days to evaluate the effect of COL3A1-knockwon CAF-D on the Matrigel invasion of 3D FaDu spheroid.

### 4.12. Statistical Analysis

Differences between groups were evaluated with the parametric two-tailed non-paired *t* test. Analyses were performed using Origin v.8.0 software (OriginLab, Northampton, MA, USA), and *p* values ≤ 0.05 were considered statistically significant.

## 5. Conclusions

CAFs derived from some HNSCC patients is less effective in promoting Cancer progression than CAF from other Patients. CAFs that overexpress *COL3A1* and *COL6A6* are presumed to be less effective in promoting cancer progression compared to those that show low overexpression. Further studies are necessary to confirm the molecular mechanisms underlying the effect of *COL3A1* and *COL6A6* expression in CAF on ECM remodeling. Particularly, studies on the effect of each collagen subtype structure on cancer cell migration or invasion should be conducted. Our results are expected to provide important information for the prognosis and drug target development of HNSCC in the future.

## Data Availability

The data presented in this study are available on request from the corresponding author.

## Supplementary Materials

The following is available online at https://www.mdpi.com/2072-6694/13/4/654/s1: Table S1: Characteristics of patients with HNSCC, Table S2: Functional annotation of genes differentially expressed in CAF-P and CAF-D fibroblasts, Table S3: Differential expression of collagen mRNAs in CAF cells; Table S4. David functional analysis of collagen protein, Figure S1: Effect of CAF-P and CAF-D on 2-dimensional Matrigel invasion of HNSCC cells, Figure S2: Effect of collagen knockdown-CAF-D on HNSCC cell invasion.

## Abbreviations

HNSCC	head and neck squamous cell carcinoma
OSCC	oral squamous cell carcinoma
ECM	extracellular matrix
CAF	cancer-associated fibroblast
CM	conditioned medium
