# Supplementary material for: Cancer-Associated Fibroblast Subgroups Showing Differential Promoting Effect on HNSCC Progression
Source: Cancers (Basel). 2021 Feb 6;13(4):654. doi: 10.3390/cancers13040654 (PMC7915931; doi:10.3390/cancers13040654)
Supplement: Supplementary file 1 [file cancers-13-00654-s001.pdf]

Supplementary Materials: Cancer-Associated Fibroblast Subgroups Showing Differential Promoting Effect on HNSCC Progression

Soo Hyun Kang, Su Young Oh, Heon-Jin Lee, Tae-Geon Kwon, Jin-Wook Kim, Sung-Tak Lee, So-Young Choi and Su-Hyung Hong

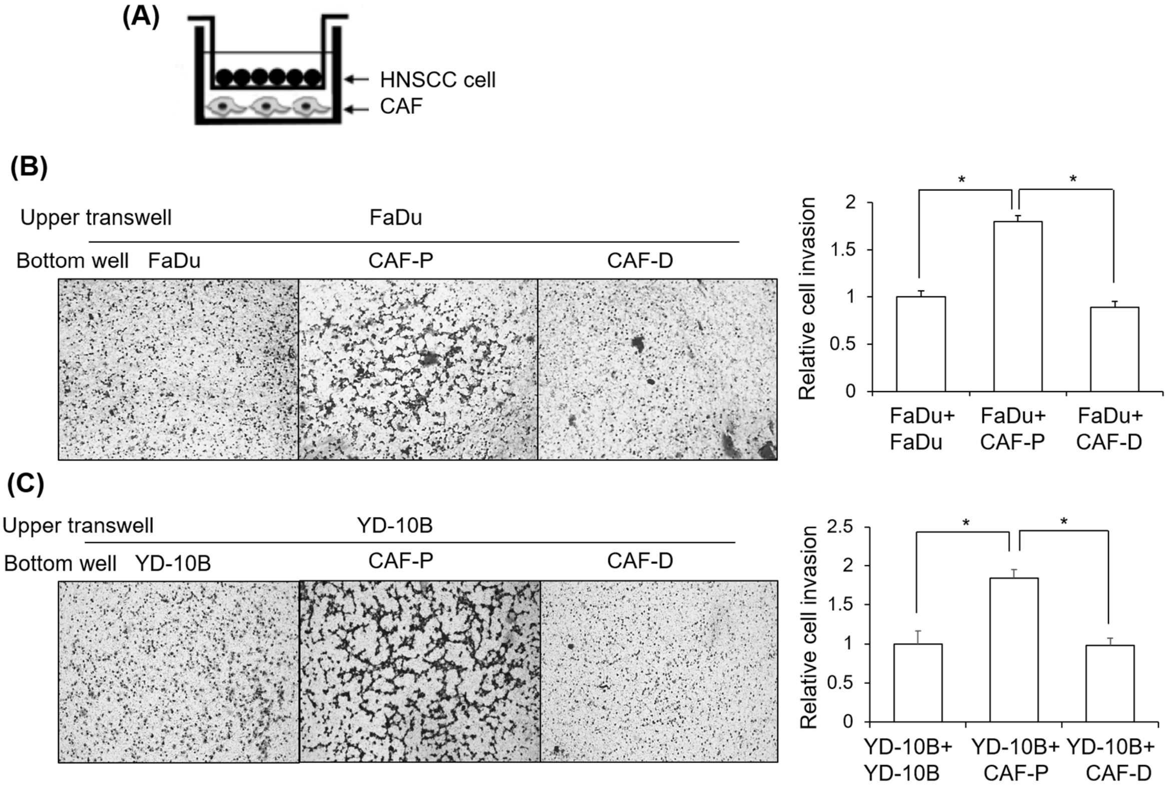

Figure S1. Effect of CAF-P and CAF-D on 2-dimensional Matrigel invasion of HNSCC cells. (**A**) CAFs were seeded in the bottom well of the 24-well plate. HNSCC cells were added to the Matrigel-coated transwell. (**B, C**) After culturing for 48–72 h, HNSCC cells in the transwell chamber were stained with crystal violet and those that had migrated to the lower surface of the transwell chamber were counted (5× magnification). The cell invasion index was calculated as the number of invaded cells between the CAF-P or CAF-D and FaDu control. Results represent the mean ± standard deviation of 3 experiments (* $p < 0.01$).

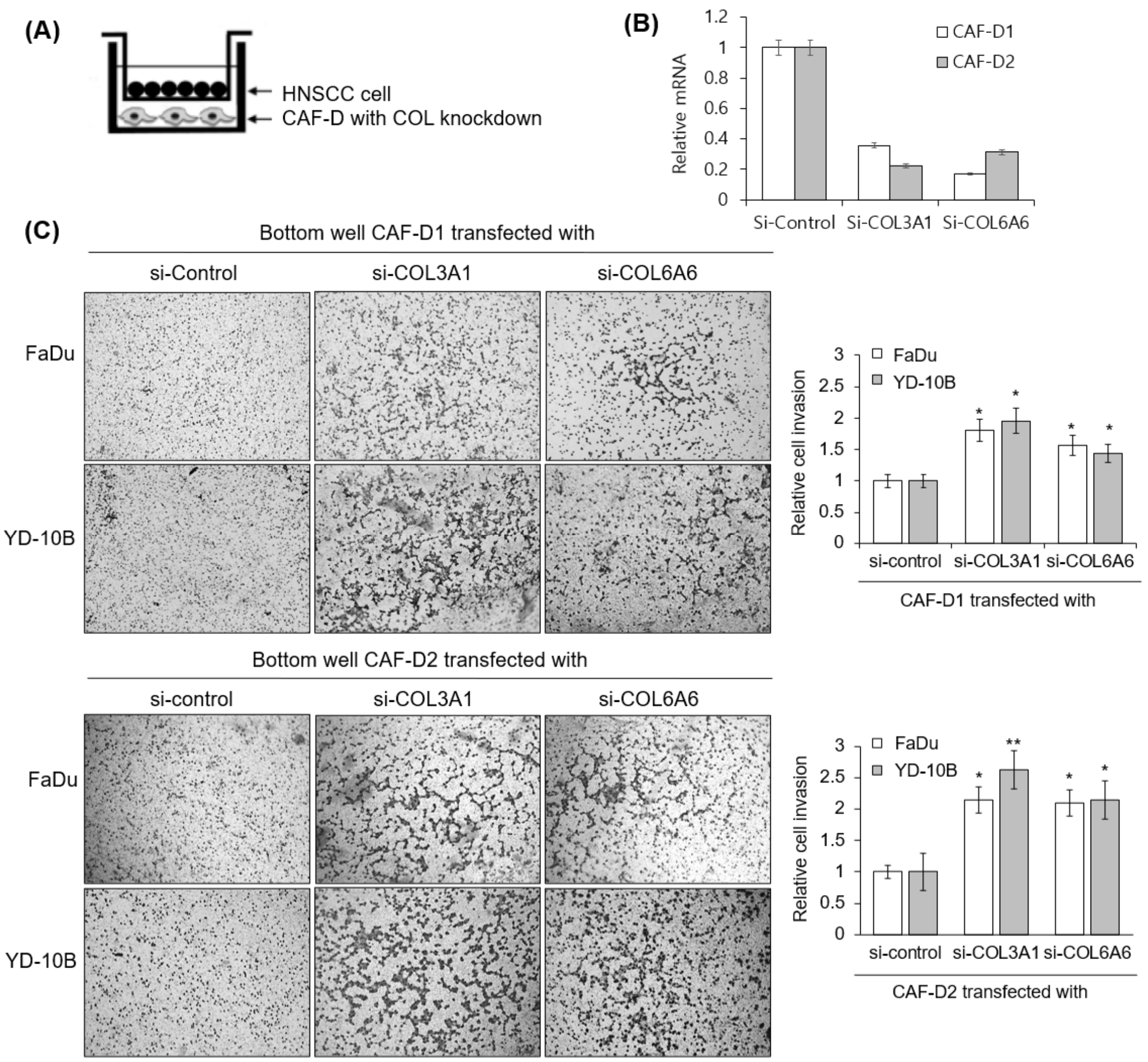

Figure S2. Effect of collagen knockdown-CAF-D on HNSCC cell invasion. (**A**) Primary CAF-D cells were transfected with si*COL3A1* or siCOL6A6. After 6 h, CAF-D cells were co-cultured with FaDu and YD-10B HNSCC cells for Matrigel invasion under the transwell system for 2 days. (**B**) At 2 days after transfecting each collagen siRNA, qPCR was performed to check the knockdown efficiency. (**C**) siRNA-transfected CAF-D cells were seeded in a 24-well plate. HNSCC cells were added to Matrigel-coated transwell chamber. The cells were stained with 0.2% crystal violet in 10% ethanol after culturing for 48–72 h, and cells that had migrated to the lower surface of the chamber were counted. The invasion index was calculated as the fold change in the number of invaded cells in the experimental group compared with that in the control group with scrambled siRNA. Results represent the mean ± standard deviation of 3 experiments (* $p < 0.05$, ** $p < 0.01$).

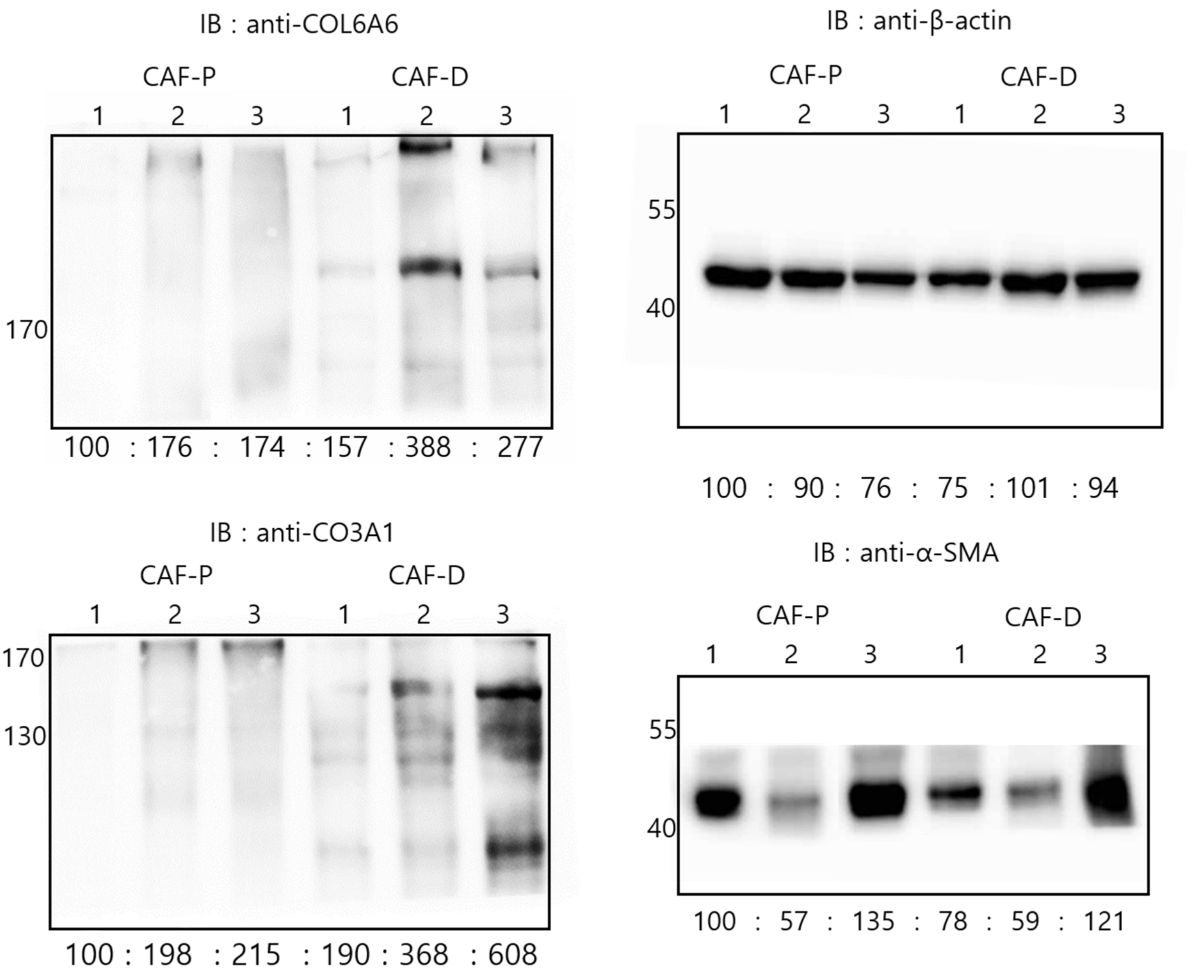

Figure S3. Raw image of western blots. Blots were cut and used to confirm the protein bands of β-actin as an internal control and α-SMA with the target proteins, respectively, from the same blot.

Table S1. Characteristics of patients with HNSCC.

CAF	Age	Sex	Primary Site	Differentiation	Lymph Node Meta	TMN	Stage	Smoking	Drinking
P	38	F	Gingiva	Well	N	T4aN0M0	4A	N	N
	48	F	Buccal mucosa	Moderate	Y	T1N1M0	3	Y	Y
	76	M	Gingival area	Moderate	N	T4N0M0	4	N	N
D	53	M	Mouth floor	Well	N	T1N0M0	1	Y	Y
	64	M	Mouth floor	Moderate	N	T2N0M0	2	N	N
	47	M	Ventral surface of tongue	Well	N	T1N0M0	1	Y	N

Table S2. Functional annotation of genes differentially expressed in CAF-P and CAF-D fibroblasts.

Category	Term	Count	*p*-Value	Genes
GOTERM-BP Biological Process	GO:0030574~collagen catabolic process	3	0.015	COL3A1, ADAMTS14, COL6A6
	GO:0032211~negative regulation of telomere maintenance via telomerase	2	0.034	HNRNPU, HNRNPA1
GOTERM_CC Cellular Compartment	GO:0005654~nucleoplasm	16	0.018	EED, FUS, HIST1H2BL, GLIS3, HNRNPU, ZBTB20, NR3C2, SEL1L3, PM20D2, RAD51D, DMTF1, POLR1A, AGO2, HNRNPA1, UTRN, FKBP5
	GO:0005581~collagen trimer	2	0.043	COL3A1, COL6A6
GOTERM_MF Molecular Function	GO:0003676~nucleic acid binding	9	0.006	ZNF681, ZNF250, FUS, AGO2, GLIS3, ZBTB20, ZNF138, HNRNPA1, ZNF431
	GO:0046872~metal ion binding	13	0.011	SMG1, ZNF681, ZNF250, GLIS3, ACSM2A, ZBTB20, ADAM20, PDP1, COL3A1, AGAP6, AGO2, ZNF138, ZNF431
	GO:0005178~integrin binding	3	0.036	COL3A1, DST, UTRN
	GO:0003677~DNA binding	10	0.044	RAD51D, ZNF681, DMTF1, ZNF250, FUS, POLR1A, HIST1H2BL, GLIS3, HNRNPU, ZBTB20
	GO:0005178~integrin binding	8	0.046	ADAMTS14, TRIM52, FUS, POLR1A, CHORDC1, PGGT1B, UTRN, NR3C2

Annotation of 99 mRNAs performed by utilizing the DAVID Functional Analysis website. (Fold change CAF-D/CAF-P > 1.75 or <0.65, and $P < 0.05$).

Table S3. Differential expression of collagen mRNAs in CAF cells.

Probe Set ID	Gene Symbol	Gene Name	Fold Change (CAF-D/CAF-P)	*p*-Value
16888610	COL3A1	collagen type III alpha 1 chain	2.362	0.041
16945543	COL6A6	collagen type VI alpha 6 chain	2.156	0.050
16978896	COL25A1	collagen type XXV alpha 1 chain	1.434	0.030
17049717	COL26A1	collagen type XXVI alpha 1 chain	1.488	0.004

Table S4. David functional analysis of collagen protein.

ID	Species	GOTERM_CC_DIRECT
COL3A1	Homo sapiens	GO:0005576~extracellular region
COL6A6	Homo sapiens	GO:0005576~extracellular region
COL25A1	Homo sapiens	GO:0005576~extracellular region
COL26A1	Homo sapiens	GO:0005576~extracellular region
**ID**	**Species**	**GOTERM_BP_DIRECT**
COL3A1	Homo sapiens	GO:0007160~cell-matrix adhesion ,GO:0007179~transforming growth factor beta receptor signaling pathway, GO:0007229~integrin-mediated signaling pathway
COL6A6	Homo sapiens	GO:0007155~cell adhesion, GO:0030574~collagen catabolic process,
COL25A1	Homo sapiens	GO:0030574~collagen catabolic process, GO:0060385~axonogenesis involved in innervation,
COL26A1	Homo sapiens	GO:0010811~positive regulation of cell-substrate adhesion, GO:0030574~collagen catabolic process,
**ID**	**Species**	**KEGG_PATHWAY**
COL3A1	Homo sapiens	hsa04151:PI3K-Akt signaling pathway, hsa04510:Focal adhesion, hsa04512:ECM-receptor interaction, hsa04611:Platelet activation, hsa05146:Amoebiasis,
COL6A6	Homo sapiens	hsa04151:PI3K-Akt signaling pathway,hsa04510:Focal adhesion,hsa04512:ECM-receptor interaction ,hsa04974:Protein digestion and absorption,
